# Downregulation of alcohol dehydrogenase family proteins suggests disrupted retinol metabolism in neonatal respiratory distress syndrome: an umbilical cord blood proteomics study

**DOI:** 10.3389/fped.2026.1949593

**Published:** 2026-09-29

**Authors:** Jing Wang, Ying Wei, Xuan Kan, Qianqian Du

**Affiliations:** Department of Pediatrics, Tianjin Medical University General Hospital, Tianjin, China

**Keywords:** alcohol dehydrogenase, neonatal respiratory distress syndrome, pathogenesis, proteomics, retinol metabolism, umbilical cord blood

## Abstract

**Objective:**

Neonatal respiratory distress syndrome (NRDS) is a prevalent complication in preterm infants, characterized by severe pulmonary dysfunction and elevated mortality rates. To systematically profile the umbilical cord blood proteome in neonates with NRDS, aiming to identify and explore their potential pathological mechanisms.

**Methods:**

A case-control study was conducted, enrolling 60 preterm infants (< 34 weeks gestational) born between November 2024 and October 2025, including 32 NRDS cases and 28 matched controls. Umbilical cord blood plasma was analyzed via nanomaterial-based low-abundance protein enrichment (n_LAPE) and liquid chromatography-tandem mass spectrometry (LC-MS/MS) in data-independent acquisition mass spectrometry (DIA) mode. Differentially expressed proteins (DEPs) were identified using the Limma package. Bioinformatics analyses included Gene Ontology (GO), KEGG pathway enrichment, protein-protein interaction (PPI) network construction, and gene set enrichment analysis (GSEA). The Least Absolute Shrinkage and Selection Operator (LASSO) regression and Random Forest algorithm were employed to screen for core proteins and evaluate their discriminatory potential.

**Results:**

A total of 7,529 high-confidence proteins were identified (5,090 passed quantification filters and entered differential expression analysis), with 157 DEPs between groups. These DEPs were significantly enriched in retinol metabolism, and complement and coagulation cascades. Both PPI network and GSEA analyses consistently highlighted the downregulation of alcohol dehydrogenase (ADH) family proteins (ADH1A, ADH1B, ADH4, ADH6) as the most prominent differential feature. An exploratory model based on five proteins, including ADH4, yielded AUCs of 0.982 (95% CI 0.95–1.00) and 0.838 (95% CI 0.63–1.00) in the training and test sets, respectively.

**Conclusion:**

The umbilical cord blood proteome of NRDS neonates is significantly disrupted, characterized by the downregulation of ADH family proteins. This suggests that ADH downregulation may contribute to NRDS pathogenesis by interfering with retinol metabolism, offering a novel mechanistic perspective. While the exploratory discriminatory model shows promise, it requires validation in large-scale prospective cohorts.

## 1 Introduction

Neonatal respiratory distress syndrome (NRDS) persists as a primary cause of morbidity and mortality among preterm infants, driven fundamentally by pulmonary surfactant (PS) deficiency and structural lung immaturity (1). NRDS affects approximately 30% of infants born at 28–34 weeks of gestation and approximately 60% of those born before 28 weeks, and the neonatal mortality rate in China was 3.1 per 1,000 live births in 2021; a nationwide survey of 394 Chinese hospitals (the MUNICH survey) further reported substantial disparities in resource availability and guideline adherence for RDS management across hospitals (2). Although the administration of exogenous PS has markedly improved clinical outcomes, the upstream signaling cascades governing PS synthesis and lung maturation remain incompletely characterized (3, 4). Furthermore, current diagnostic frameworks depend heavily on postnatal clinical manifestations, imaging, and blood gas analysis, which often fail to capture the immediate, molecular-level pathophysiological perturbations occurring at birth (5). Consequently, a systems biology approach is essential to unravel the molecular architecture of NRDS at disease onset, thereby illuminating its pathophysiology and revealing novel therapeutic targets.

Umbilical cord blood, obtainable at delivery, is a key biological specimen reflecting the late intrauterine fetal state with minimal immediate extrauterine influence, providing a unique window into early molecular events of fetal-origin diseases like NRDS (6). Proteins, as direct executors of biological functions, reflect the most direct manifestations of disease mechanisms. However, the detection of low-abundance functional proteins in plasma is severely hampered by high-abundance proteins, limiting the depth of proteomics research (7). The nanomaterial-based low-abundance protein enrichment (n_LAPE) technique, which indiscriminately enriches low-abundance proteins, effectively overcomes this bottleneck, enabling efficient enrichment and identification of functional low-abundance plasma proteins (8) and facilitating in-depth mechanistic exploration. Because many functionally critical regulators, including transcription factors, cytokines, and metabolic enzymes such as the ADH family, are present at low abundance in plasma, their enrichment is essential for capturing disease-relevant proteomic changes. Although previous peptidomic and targeted proteomic studies have begun to address NRDS in cord blood (6, 9), systematic research at the intact proteome level focusing on NRDS pathogenesis, particularly pathways related to PS metabolism, remains relatively limited.

Retinol (vitamin A) metabolism is closely linked to fetal lung maturation: retinol is oxidized by ADH family enzymes to retinaldehyde and further to retinoic acid, which regulates alveolar type II epithelial cell differentiation and pulmonary surfactant synthesis (10). The peroxisome proliferator-activated receptor (PPAR) signaling pathway, particularly PPAR*γ*, coordinates lipid metabolism and alveolar development (11). These interlinked pathways therefore provide a plausible mechanistic framework linking cord blood proteomic changes to NRDS pathogenesis.

This study utilized n_LAPE-DIA proteomics to conduct a comprehensive analysis of umbilical cord blood plasma obtained from infants with NRDS and healthy preterm controls. The specific objectives were to: 1) systematically characterize the NRDS-associated differential proteomic landscape and elucidate its underlying molecular mechanisms; and 2) secondarily, to evaluate the discriminatory potential of key DEPs, thereby providing candidate biomarkers for future translational research. The primary objective was mechanistic hypothesis generation, while biomarker evaluation was a secondary, exploratory aim.

## 2 Materials and methods

### 2.1 Study design and patient population

This single-center case-control study was conducted at Tianjin Medical University General Hospital. We consecutively enrolled preterm infants born between November 2024 and October 2025, with a gestational age of less than 34 weeks, who were admitted to the Neonatal Intensive Care Unit (NICU). Participants were stratified into two groups based on the presence of NRDS: the NRDS group (*n* = 32), diagnosed in accordance with the European Consensus Guidelines on the Management of Respiratory Distress Syndrome (1), and the control group (*n* = 28), comprising gestation-matched preterm infants born during the same period who did not develop NRDS or other severe complications necessitating respiratory support. Exclusion criteria encompassed major congenital anomalies, confirmed intrauterine infection, severe birth asphyxia, and maternal history of severe preeclampsia or uncontrolled gestational diabetes. The sample size was determined on the basis of feasibility, consistent with the exploratory nature of the study; no formal power calculation was performed, and the statistical power for detecting modest differences is therefore limited. The groups were balanced for the major confounders of NRDS, including gestational age, birth weight, sex, delivery mode, and maternal pregnancy complications (Table 1); nevertheless, residual confounding by unmeasured perinatal factors cannot be fully excluded. Inclusion and exclusion criteria were defined according to the European Consensus Guidelines on the Management of Respiratory Distress Syndrome (1) and the clinical practice standards of our center.

**Table 1:** Comparison of baseline clinical characteristics between the NRDS and control groups.

Characteristic	NRDS (*n* = 32)	Control (*n* = 28)	Statistic	*P*-value
Maternal Factors				
Hypertensive disorders of pregnancy, *n* (%)	17 (53.1%)	9 (32.1%)	*χ*^2^ = 2.677	0.102
Gestational diabetes, *n* (%)	15 (46.9%)	16 (57.1%)	χ^2^ = 0.630	0.427
Antenatal corticosteroids, *n* (%)	23 (71.9%)	21 (75.0%)	χ^2^ = 0.075	0.785
Cesarean delivery, *n* (%)	18 (56.3%)	13 (46.4%)	χ^2^ = 0.577	0.448
Neonatal Factors				
Male, *n* (%)	21 (65.6%)	15 (53.6%)	χ^2^ = 0.904	0.342
Birth weight, g, mean ± SD	1565.0 ± 241.8	1639.1 ± 199.7	t = −1.283	0.205
Gestational age, weeks, mean ± SD	30.97 ± 1.09	31.39 ± 1.26	t = −1.375	0.175
Duration of non-invasive support, h, median (IQR)	127.5 (16.5, 251.3)	0.0 (0.0, 9.5)	Z = −6.019	<0.001​

Data are presented as n (%), mean ± SD, or median (IQR). P-values were derived from Chi-square test, independent samples t-test, or Mann–Whitney U test, as appropriate. Apgar score, multiple gestation, chorioamnionitis, and premature rupture of membranes were not recorded in the clinical database and therefore could not be included in Table 1.

### 2.2 Sample collection and proteomic analysis

Immediately after delivery, approximately 5 mL of umbilical cord blood was collected aseptically into EDTA tubes. Plasma was separated by centrifugation at 1,600×g for 15 min at 4 °C, aliquoted, and stored at −80 °C. Low-abundance proteins were enriched from plasma using the n_LAPE kit (Putian Biotech, SR005). Enriched proteins were digested with trypsin, and the resulting peptides were desalted prior to LC-MS/MS analysis. Chromatographic separation was performed using a C18 reversed-phase column. Mass spectrometric analysis was conducted in DIA mode with a scan range of m/z 150−2,000. A pooled quality control sample, created by mixing aliquots from all samples, was analyzed every 20 runs to monitor process stability. All samples were processed in a single batch.

### 2.3 Data analysis

#### 2.3.1 Protein identification and quantification

Data were processed using DIA-NN software (v1.8.1) against the UniProt human protein database, with a false discovery rate threshold of 1%. Label-free protein quantification was performed within the DIA-NN pipeline (MaxLFQ-based intensity normalization). Proteins with >70% missing values within a comparison group were excluded, and remaining missing values were imputed with half of the protein's minimum intensity in that group.

#### 2.3.2 Differential expression analysis

The Limma package in R was used for statistical analysis of normalized protein expression data. Proteins with |log2(fold change)| > 0.585 and a nominal *P*-value < 0.05 were defined as DEPs. Benjamini-Hochberg false discovery rate (FDR) correction was additionally applied; however, no protein reached FDR < 0.05, so DEPs are reported at the nominal level and interpreted as exploratory hypothesis-generating findings.

#### 2.3.3 Bioinformatics analysis

DEPs were subjected to GO and KEGG pathway enrichment analyses. A PPI network was constructed using the STRING database with standard parameters (confidence score threshold of 0.4, i.e., STRING medium confidence); core functional modules were identified within the STRING network. GSEA was performed to assess the distribution of the global protein expression profile across KEGG pathways.

#### 2.3.4 Clinical data analysis

Statistical analysis was performed using SPSS 26.0. Normally distributed continuous data are presented as mean ± standard deviation and compared using the independent samples t-test; non-normally distributed data are presented as median (interquartile range) and compared using the Mann–Whitney U test. Categorical data are presented as number (percentage) and compared using the Chi-square test.

#### 2.3.5 Exploratory evaluation of discriminatory potential

First, univariate receiver operating characteristic (ROC) curve analysis was performed on DEPs, and the top 10 proteins with an AUC > 0.7 were selected as candidates. Least Absolute Shrinkage and Selection Operator (LASSO) regression with 20-fold cross-validation was then applied for dimension reduction and selection. Based on the selected core proteins, the total cohort was randomly split into a training set and a test set in a 7:3 ratio. A Random Forest classifier was built on the training set (with 10-fold cross-validation), and its performance was evaluated on the independent test set, with the AUC, sensitivity, and specificity as the primary metrics. This analysis is explicitly derivative and exploratory, aimed at hypothesis generation rather than diagnostic model development; Random Forest hyperparameters were kept at package defaults, and full parameter details are available from the corresponding author upon request.

## 3 Results

### 3.1 Clinical characteristics of the study population

Sixty preterm infants were included (NRDS: *n* = 32; Control: *n* = 28). No significant differences were observed between the two groups in terms of sex, birth weight, gestational age, delivery mode, or maternal pregnancy complications (all *P* > 0.05), indicating balanced baseline characteristics (Table 1). The duration of postnatal respiratory support was significantly longer in the NRDS group than in the control group (median [IQR]: 127.5 [16.5, 251.3] h vs. 0.0 [0.0, 9.5] h, *P* < 0.01).

### 3.2 Global proteomic profiling of umbilical cord blood

The n_LAPE-DIA platform identified 7,529 high-confidence non-redundant proteins across all samples (of which 5,090 passed quantification filters and were included in differential expression analysis), with no significant difference in the number of proteins detected between groups (Figure 1A). Using the thresholds of |log2FC| > 0.585 and *P* < 0.05, 157 DEPs were identified, with 116 upregulated and 41 downregulated in the NRDS group (Figure 1B). Principal component analysis based on all 5,090 quantified proteins showed substantial overlap between the NRDS and control groups (Figure 1C, revised), with only a weak central tendency shift (Dim1 = 35.9%, Dim2 = 7.8%), indicating that the groups were not cleanly separated and consistent with the exploratory nature of the findings.

**Figure 1:**
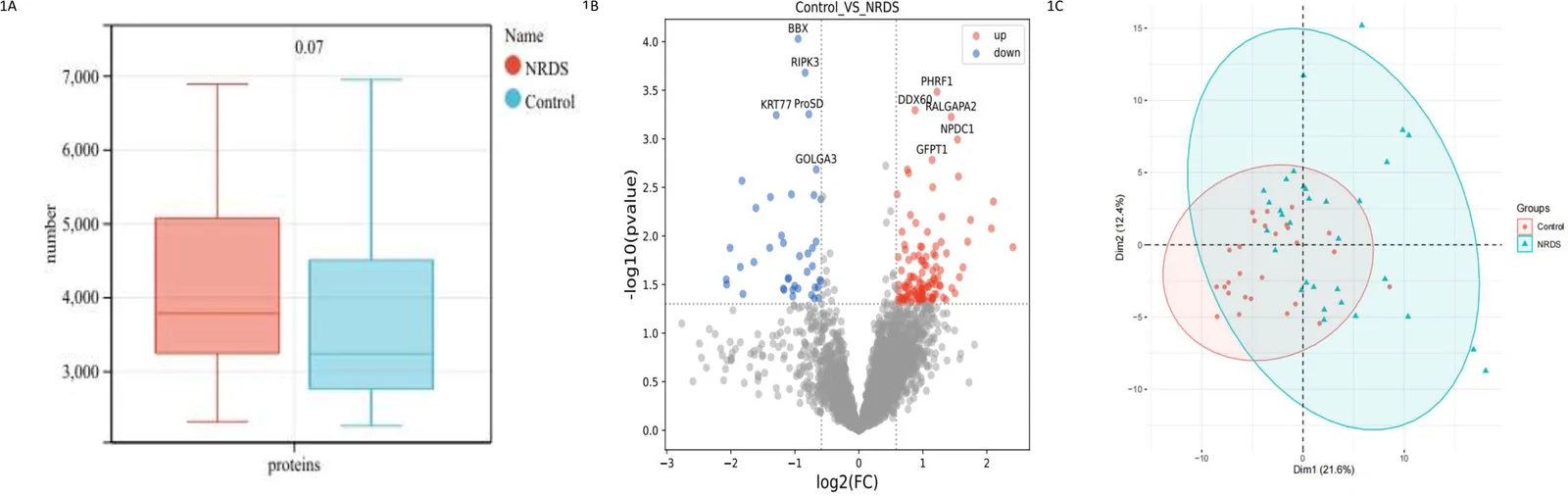
Proteomic profiling and identification of differentially expressed proteins (DEPs). **(A)** Number of proteins identified in the Neonatal Respiratory Distress Syndrome (NRDS) and control groups. No significant difference was observed (*P* > 0.05). **(B)** Volcano plot displaying the DEPs between groups. Red dots represent upregulated proteins, blue dots represent downregulated proteins and gray dots represent non-significant proteins. **(C)** Principal Component Analysis (PCA) score plot based on all 5,090 quantified proteins, showing substantial overlap between the NRDS and control groups (Dim1 = 35.9%, Dim2 = 7.8%) with only a weak central tendency shift. The 157-DEPs-only PCA is provided as Supplementary Figure S1 for transparency.

### 3.3 Functional enrichment of differentially expressed proteins

KEGG pathway enrichment analysis revealed that DEPs were enriched in pathways including “Retinol metabolism” (gene ratio 4/78; BH-adjusted *P* = 0.014), “Tyrosine metabolism” (4/78; adjusted *P* = 0.044), “Drug metabolism” (4/78; adjusted *P* = 0.044), “Metabolism of xenobiotics by cytochrome P450” (4/78; adjusted *P* = 0.044), and “Alcoholic liver disease” (7/78; adjusted *P* = 0.048) (Figure 2A; Supplementary Table S2). “Complement and coagulation cascades” was enriched at the nominal level (7/78; *P* = 0.003) but did not reach the BH-adjusted significance threshold (adjusted *P* = 0.067).

**Figure 2:**
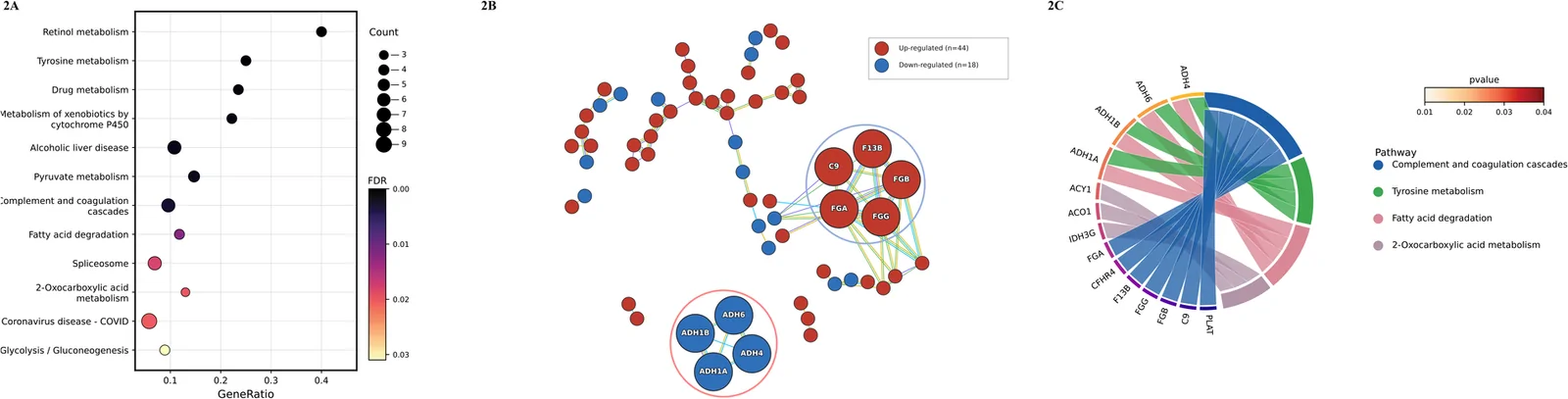
Functional enrichment and protein-protein interaction (PPI) network of DEPs. **(A)** Kyoto Encyclopedia of Genes and Genomes (KEGG) pathway enrichment analysis of DEPs. The top 10 significantly enriched pathways are shown. The dot size represents the number of DEPs in the pathway, and the color represents the -log10(*P*-value). **(B)** PPI network of DEPs constructed using the STRING database. Two core functional modules are highlighted: the Alcohol Dehydrogenase (ADH) family module (red nodes) and the Fibrinogen/Coagulation module (blue nodes). Edge thickness indicates the strength of interaction evidence. **(C)** Pathway analysis of proteins within the two core PPI modules confirms enrichment in “Tyrosine metabolism” and “Complement and coagulation cascades”.

### 3.4 Global protein expression profiling identifies ADH family as the core differential node

GSEA based on KEGG pathways indicated significant alterations in the global protein expression profile associated with pathways such as “PPAR signaling pathway”, “Tyrosine metabolism”, and “Starch and sucrose metabolism” (Figure 3A). Integrating proteins from all significantly enriched pathways identified 9 frequently occurring key proteins (Figure 3B). Venn diagram analysis showed that 4 of these 9 proteins overlapped with the 157 DEPs list, and all belonged to the ADH family (ADH1A, ADH1B, ADH4, ADH6) (Figure 3C). The expression levels of these 4 ADH proteins were all significantly downregulated in the NRDS group (Table 2). This finding, corroborated by the PPI network analysis, suggests the downregulation of ADH family proteins as a leading candidate differential feature in the NRDS umbilical cord blood proteome.

**Figure 3:**
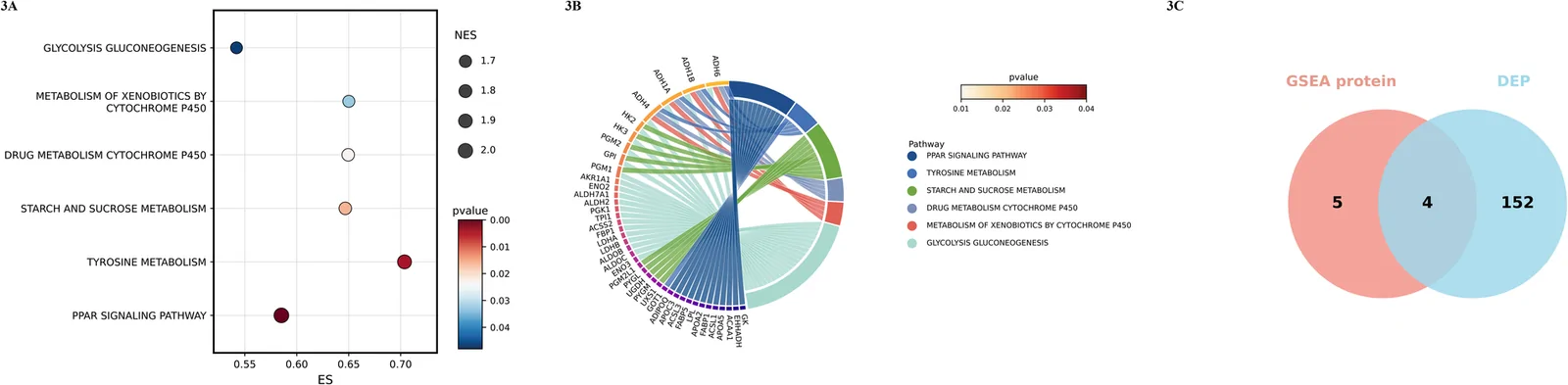
Identification of Core Differential Pathways and Proteins via Gene Set Enrichment Analysis (GSEA). (A) GSEA plot showing the most significantly altered KEGG pathways based on the global protein expression profile. The normalized enrichment score (NES) and false discovery rate (FDR) are indicated. (B) Circos plot illustrating the mapping of key enriched proteins to significantly enriched KEGG pathways (FDR < 0.25) identified by GSEA. (C) Venn diagram illustrating the overlap between the 9 frequently occurring key proteins from GSEA and the 157 DEPs. The 4 overlapping proteins (ADH1A, ADH1B, ADH4, ADH6) constitute the core differential protein set.

**Table 2:** Information on core differentially expressed proteins.

Protein	Protein description	Control group (Intensity)	NRDS group (Intensity)	log2(FC)	*P*-value
ADH1B	Alcohol dehydrogenase 1B	7,476.2	3,492.4	−1.09	0.002
ADH1A	Alcohol dehydrogenase 1A	19,243.9	10,125.6	−0.92	0.017
ADH4	All-trans-retinol dehydrogenase [NAD(+)]	47,135.1	13,312.0	−1.83	<0.001
ADH6	Alcohol dehydrogenase 6	7,639.6	3,396.4	−1.17	0.005

Protein intensity values are presented as the average normalized intensity in each group. Statistical significance was determined by the Limma package.

### 3.5 Exploratory discriminatory analysis based on core proteins

Given the central role of the ADH family in the mechanistic analysis, we further evaluated the inter-group discriminatory potential of key proteins. The top 10 candidate proteins with AUC > 0.7 were selected via univariate ROC analysis (Figure 4A). LASSO regression screening yielded a final panel of 5 core proteins (frequency > 0.8): RALGAPA2, KRT77, NPDC1, PHRF1, and ADH4, which includes the core mechanistic protein ADH4. Evaluation based on these 5 proteins using the Random Forest algorithm showed preliminary discriminatory potential in internal validation: the AUCs were 0.982 (95% CI 0.95–1.00) and 0.838 (95% CI 0.63–1.00) in the training and independent test sets, respectively (Figure 4B). The gap between these values suggests a risk of overfitting, indicating that this finding requires validation in an independent, larger cohort.

**Figure 4:**
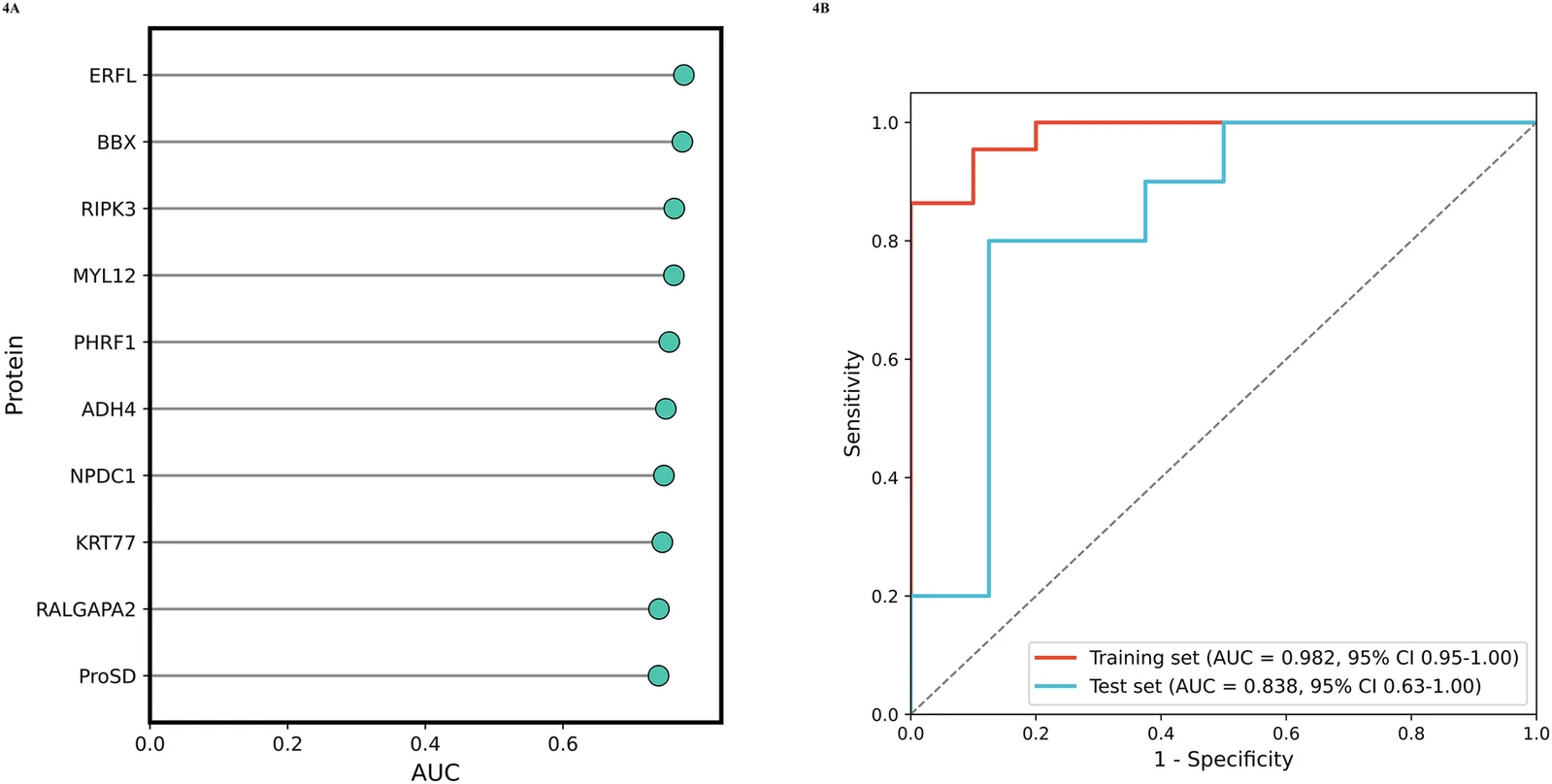
Exploratory Evaluation of the Discriminatory Potential of Core Proteins. **(A)** Univariate Receiver Operating Characteristic (ROC) curve analysis for the top 10 candidate proteins (Area Under the Curve, AUC > 0.7). The corresponding AUC for each protein is listed. **(B)** Performance of the Random Forest classifier built on the 5-protein panel (RALGAPA2, KRT77, NPDC1, PHRF1, ADH4) selected by LASSO regression. ROC curves demonstrate the model's performance in the training set AUC=0.984 and the independent test set AUC=0.8 33. AUC = 0.982 (95% CI 0.95−1.00) in the training set and 0.838 (95% CI 0.63−1.00) in the independent test set (DeLong method).

## 4 Discussion

Through deep proteomic profiling of umbilical cord blood, this study systematically revealed a distinct protein expression signature in NRDS infants and focused on the downregulation of ADH family proteins as a core finding, exploring its potential role in disease pathogenesis via disruption of retinol metabolism. The main findings are as follows: First, NRDS infants exhibit widespread proteomic disturbances in umbilical cord blood, with DEPs significantly enriched in pathways like retinol metabolism and complement and coagulation cascades. Second, multi-level bioinformatics analyses consistently identified ADH family downregulation as a candidate central molecular feature, associating it with retinol metabolism and PPAR signaling. Finally, exploratory analysis based on a key protein panel (including ADH4) suggested potential discriminatory value but with an overfitting risk, necessitating validation in an independent cohort.

The association between ADH family downregulation and NRDS pathogenesis is a major focus of this study. ADH family enzymes are crucial for catalyzing the oxidation of retinol to retinaldehyde, a precursor for retinoic acid synthesis. Retinoic acid is a key signaling molecule regulating the differentiation and maturation of alveolar type II epithelial cells and the synthesis of PS (10). The concurrent significant downregulation of ADH1A, ADH1B, ADH4, and ADH6 observed here suggests that the conversion of retinol to retinoic acid may be impaired during the late fetal stage in NRDS infants, potentially leading to attenuated downstream retinoic acid signaling and subsequently affecting alveolar type II cell function and PS production. This inference aligns with previous clinical observations of an inverse correlation between umbilical cord blood retinol levels and NRDS risk (10, 12). Furthermore, GSEA indicated an overall alteration in the PPAR signaling pathway. PPAR*γ* is a key transcription factor regulating lipid metabolism (including phospholipid synthesis for PS) and alveolar development (13). ADH downregulation, potentially associated with disrupted retinol metabolism, may interact with the PPAR signaling pathway, forming a regulatory network affecting PS synthesis and lung maturation, which presents a direction for future experimental validation. Direct studies of ADH family proteins in NRDS remain scarce; indirect evidence linking vitamin A status to neonatal respiratory outcomes (10, 11) supports the biological plausibility of our findings. In addition, reduced ADH proteins might partly reflect immature hepatic metabolism or generalized developmental immaturity rather than NRDS-specific biology, and this alternative interpretation cannot be excluded.

Our results also showed significant enrichment of DEPs and the core PPI module in the “Complement and coagulation cascades” pathway, suggesting that innate immune overactivation and coagulopathy may be another feature of early NRDS pathophysiology. Excessive complement activation can mediate lung tissue inflammatory injury (14, 15), while coagulation dysfunction may contribute to micro thrombosis, impairing alveolar capillary perfusion (16). These echoes reports of elevated inflammatory cytokine levels in NRDS infants (17, 18), supporting the potential involvement of the “inflammation-coagulation” cascade in disease progression, possibly interacting with the metabolic disturbances described above to form a complex pathological network.

Beyond mechanistic exploration, we conducted a preliminary evaluation of the discriminatory potential of key DEPs. The 5-core-protein panel selected by LASSO regression included ADH4, consistent with the core mechanistic finding. While showing preliminary discriminatory potential in internal validation, the notable gap between training and test set AUCs (0.984 vs. 0.833 indicates a risk of overfitting (19). It must be emphasized that the primary aim of this study was hypothesis generation regarding mechanism; this analysis is purely derivative and exploratory, intended only to suggest that differential expression of key proteins like ADH holds potential for translation into discriminatory biomarkers and to provide specific targets for subsequent functional studies. Its discriminatory performance is far from established and requires rigorous validation in a well-designed, prospective, large-scale cohort study.

The strength of this study lies in the application of the n_LAPE technique, which significantly enhanced the detection depth of low-abundance proteins in umbilical cord blood (20), enabling the discovery of mechanism-related proteins like the ADH family that are low in abundance but functionally important. Furthermore, the multi-level strategy integrating differential expression analysis with global gene set enrichment analysis increased the robustness of identifying candidate core molecular features. This study has several limitations. First, the sample size was determined based on feasibility and may have limited statistical power. Second, the case-control design cannot completely control for all confounders. Although gestational age was not statistically different between groups, the slightly lower mean in the NRDS group may introduce potential bias. Third, this is a proteomics association study; the causal role of ADH down regulation in NRDS pathogenesis needs validation through functional gain- and loss-of-function studies in lung epithelial cell models, organoids, or animals. Fourth, the discriminatory model analysis involved a small sample size and lacked external validation, leading to over fitting risk; thus, the results should be considered only as hypothesis-generating. Fifth, while pathway analysis suggests ADH down regulation may affect PS synthesis, we did not directly measure the expression of pulmonary surfactant-associated proteins (SP-A, SP-B, SP-C, SP-D); future studies should include this evidence.

Finally, several mechanistic claims remain to be directly validated. The identified DEPs were not independently confirmed by targeted mass spectrometry (PRM) or immunoassays (ELISA/Western blot); retinol, retinaldehyde, and retinoic acid concentrations were not directly measured, so the proposed disruption of retinol metabolism is inferential; and surfactant proteins (SP-A/B/C/D) were not quantified, so the link to surfactant synthesis is indirect. Confirmation at the protein and metabolite level in an independent, larger cohort is required.

## 5 Conclusion

In conclusion, this umbilical cord blood proteomics study identifies downregulation of ADH family proteins as a candidate core molecular feature in NRDS infants. It innovatively proposes the hypothesis that this downregulation is associated with NRDS pathogenesis and may relate to disruption of the “retinol metabolism-PPAR signaling” axis, thereby affecting lung development and PS synthesis. Concurrently, exploratory analysis suggests that a protein panel including ADH4 has discriminatory potential, albeit with an overfitting risk. These findings provide preliminary, hypothesis-generating leads for in-depth functional research; any translational implications require validation in independent cohorts and mechanistic experiments.

## Data Availability

The original data contributions presented in the study are included in the article or supplementary material. Further inquiries can be directed to the corresponding author(s).
