# Supplementary material for: Downregulation of alcohol dehydrogenase family proteins suggests disrupted retinol metabolism in neonatal respiratory distress syndrome: an umbilical cord blood proteomics study
Source: Front Pediatr. 2026 Sep 29;14:1949593. doi: 10.3389/fped.2026.1949593 (PMC13658445; doi:10.3389/fped.2026.1949593)
Supplement: Supplementary file 1 [file Supplementaryfile1.docx]

**Attachment figures with captions - MS 1949593 (Frontiers in Pediatrics)**

Revision 2026-08-21 | All figures provided as attachments (300 dpi PNG unless stated); not uploaded to the system supplementary section. Full statistics: Supplementary Tables S2-S8 (see the Excel workbook in this package).

*Supplementary Figure S1. PCA score plot based on the 157 DEPs (the original submitted Fig. 1C panel), retained for transparency after Fig. 1C was replaced with the all-protein PCA.*

*FigS1 (vendor). Volcano plot of differential expression (NRDS vs Control); DEPs labelled (|log2FC| > 0.585, nominal P < 0.05; 116 up / 41 down).*

*FigS2 (vendor). KEGG enrichment dot plot for the 157 DEPs; Retinol metabolism BH-adjusted P = 0.014.*

*FigS3 (vendor). GO enrichment dot plot for the 157 DEPs.*

*FigS4 (vendor). Heatmap of the 157 DEPs across samples.*

*FigS5 (vendor). Heatmap of all 5,090 quantified proteins.*

*FigS11 (GSEA). KEGG Drug metabolism - cytochrome P450 (NES = 1.72, FDR q = 0.134).*

*FigS12 (GSEA). KEGG Glycolysis / Gluconeogenesis (NES = 1.64, FDR q = 0.200).*

*FigS13 (GSEA). KEGG Metabolism of xenobiotics by cytochrome P450 (NES = 1.65, FDR q = 0.222).*

*FigS14 (GSEA). KEGG PPAR signaling pathway (NES = 2.03, FDR q = 0.008).*

*FigS15 (GSEA). KEGG Starch and sucrose metabolism (NES = 1.72, FDR q = 0.178).*

*FigS16 (GSEA). KEGG Tyrosine metabolism (NES = 1.92, FDR q = 0.025).*
